# Supplementary material for: Probing hydrogen bond strength in liquid water by resonant inelastic X-ray scattering
Source: Nat Commun. 2019 Mar 4;10:1013. doi: 10.1038/s41467-019-08979-4 (PMC6399250; doi:10.1038/s41467-019-08979-4)
Supplement: Supplementary file 1 — Supplementary Information [file 41467_2019_8979_MOESM1_ESM.pdf]

Supplemental Information for: Probing Hydrogen Bond Strength in Liquid Water by Resonant inelastic X-ray scattering

Vinícius Vaz da Cruz*,[1] Faris Gel'mukhanov,[1,2] Sebastian Eckert,[3] Marcella Iannuzzi,[4] Emelie Ertan,[5] Annette Pietzsch,[6] Rafael C. Couto,[1] Johannes Niskanen,[7,6] Mattis Fondell,[6] Marcus Dantz,[8] Thorsten Schmitt,[8] Xingye Lu,[8] Daniel McNally,[8] Raphael M. Jay,[3] Victor Kimberg,[1,2] Alexander Föhlisch,[3,6] and Michael Odelius†[5]

[1] *Theoretical Chemistry and Biology, Royal Institute of Technology, 10691 Stockholm, Sweden**

[2] *Laboratory for Nonlinear Optics and Spectroscopy, Siberian Federal University, 660041 Krasnoyarsk, Russia*

[3] *Institut für Physik und Astronomie, Universität Potsdam, Karl-Liebknecht-Strasse 24-25, 14476 Potsdam, Germany*

[4] *Physical Chemistry Institute, University of Zürich, 8057 Zürich, Switzerland*

[5] *Department of Physics, Stockholm University, AlbaNova University Center, 10691 Stockholm Sweden*

[6] *Institute for Methods and Instrumentation in Synchrotron Radiation Research FG-ISRR, Helmholtz-Zentrum Berlin für Materialien und Energie Albert-Einstein-Strasse 15, 12489 Berlin, Germany*

[7] *Department of Physics and Astronomy, University of Turku, FI-20014, Turun yliopisto, Finland*

[8] *Research Department Synchrotron Radiation and Nanotechnology, Paul Scherrer Institut, CH-5232 Villigen PSI, Switzerland*

* vvdc@kth.se, † odelius@fysik.su.se

I. SUPPLEMENTARY FIGURES

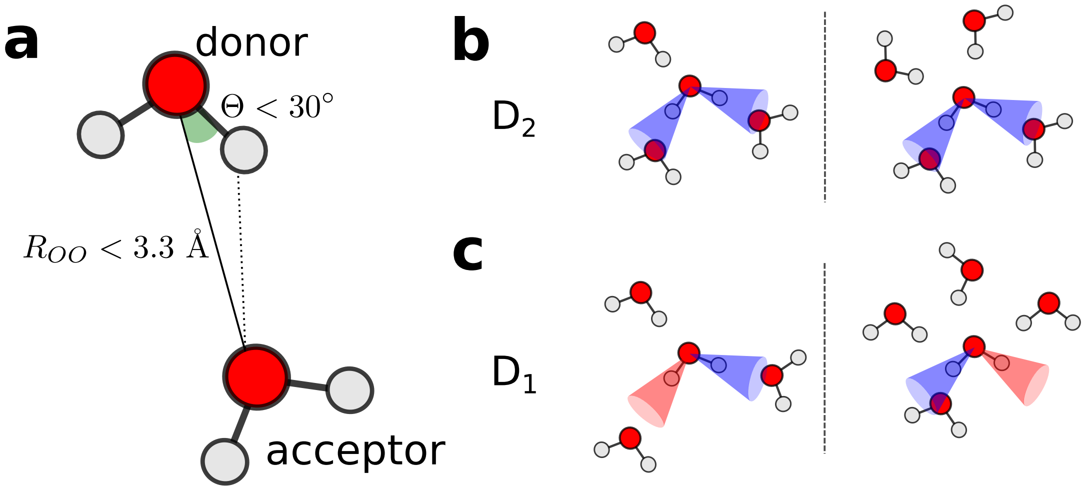

Supplementary Figure 1. **Hydrogen bond classification**
a Schematic depiction of the local HB classification employed. **b** and **c** Illustration of possible D2 and D1 structures, respectively.

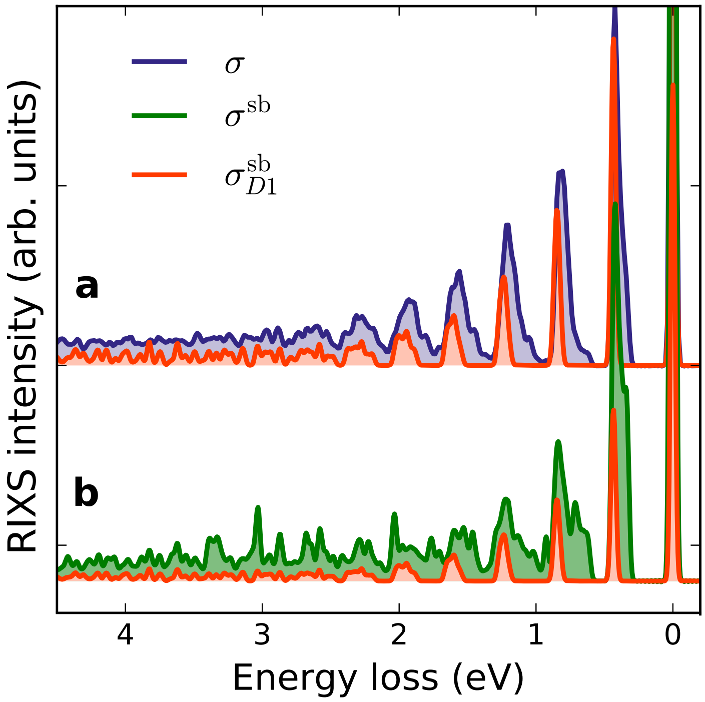

Supplementary Figure 2. **Non-validity of the single-bond model**
a Comparison of total theoretical RIXS spectrum σ (dark blue) and the RIXS composed only of the non-hydrogen bonded potentials σ^{sb}_{D1} (red) for all D1 structures (single bond calculations). **b** RIXS spectra considering a single bond model for all OH bonds and all structures in the configuration σ^{sb} (green) alongside σ^{sb}_{D1}.

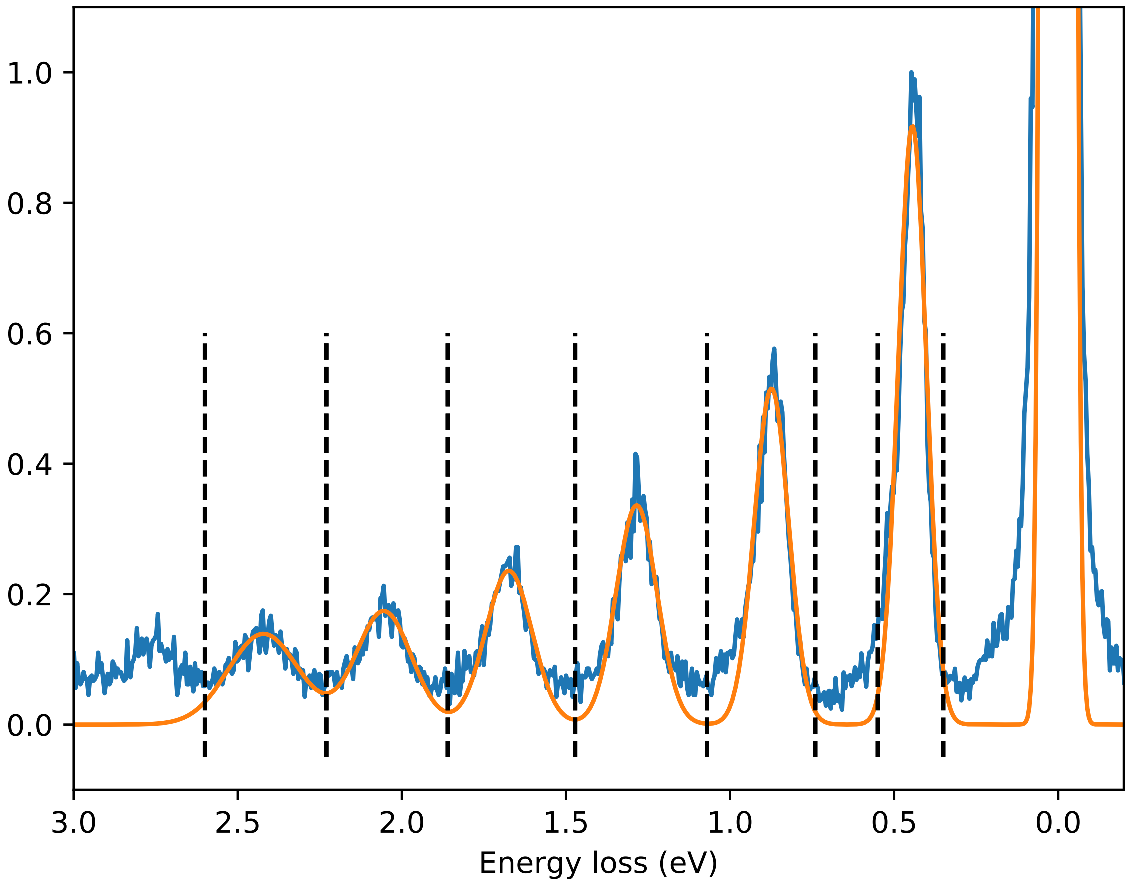

Supplementary Figure 3. **Division of the spectrum into intervals** $\Delta\epsilon_m$
Experimental RIXS spectrum and its Gaussian fit are shown by blue and orange, respectively. The division into intervals was performed using the Gaussian fit.

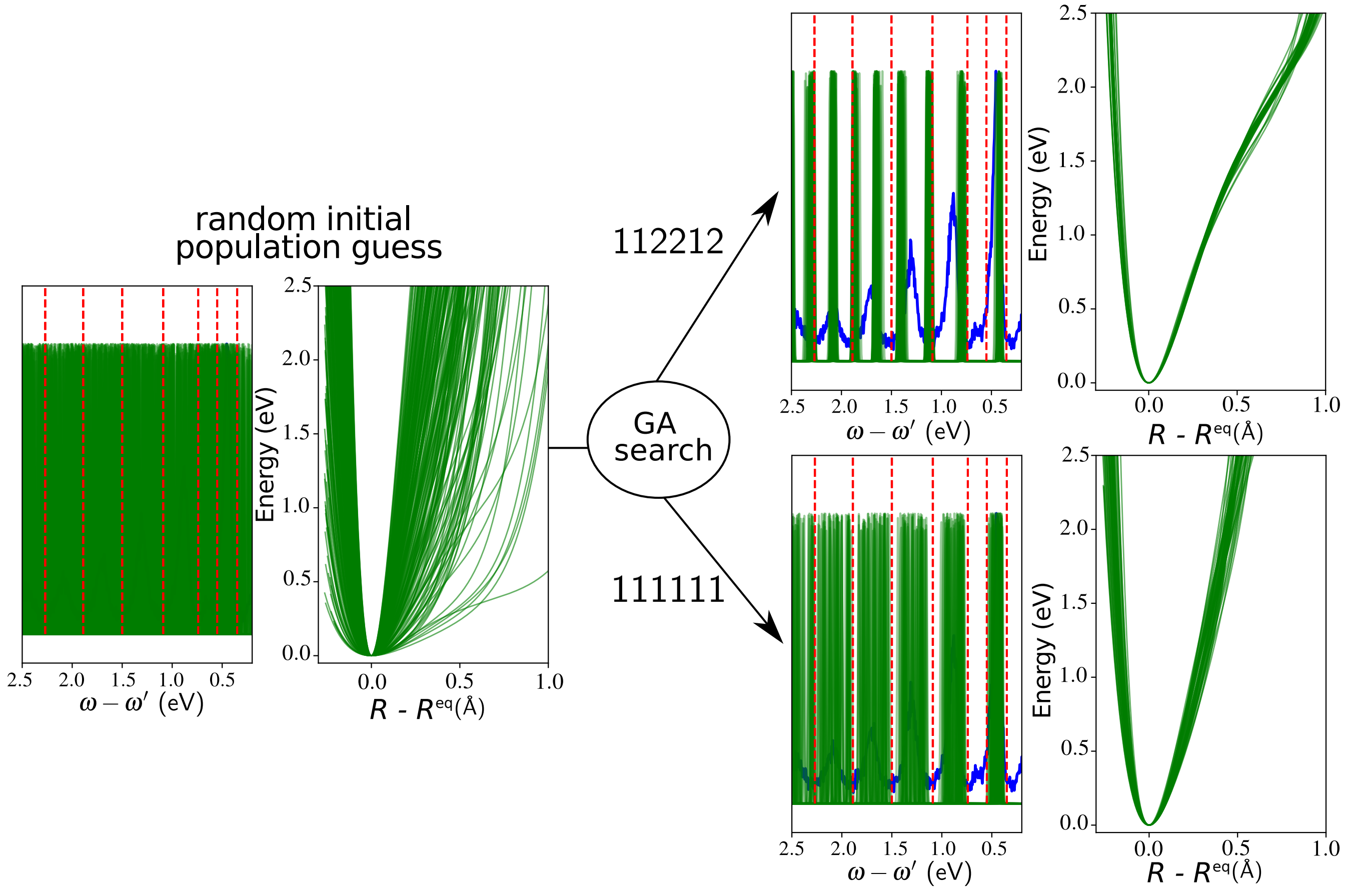

Supplementary Figure 4. **Explanation of the reconstruction of the potentials through the genetic algorithm (GA)** Left panel shows that randomly generated initial distribution of the potentials (without any constraints) leads to a quasi-continuum spectrum. Right panel displays the distribution of the OH potentials selected by GA for two individual constraints: (111111) and (112212), contained in Eqs. (2 and 3) of main text, respectively.

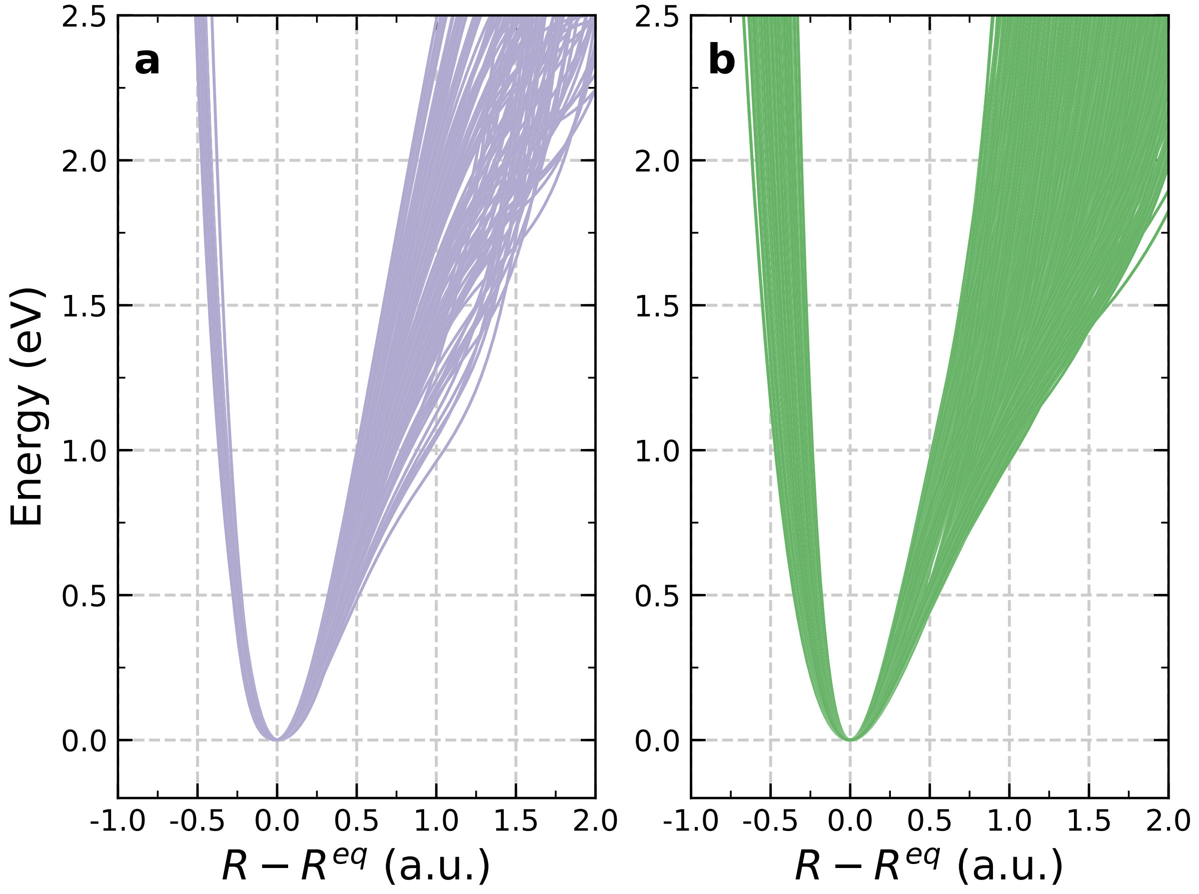

Supplementary Figure 5. **Validation of the confidence interval extraction**
a Distribution of the *ab initio* potentials. **b** Confidence interval reconstructed from the theoretical RIXS spectrum based on the potentials shown in the panel **a**.

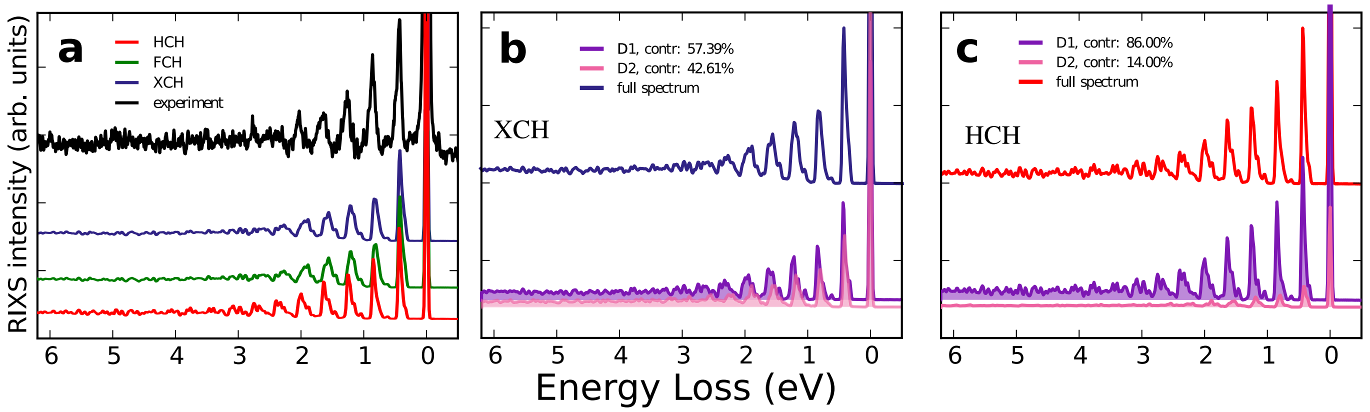

Supplementary Figure 6. **The role of the method of calculations of transition dipole moment on the RIXS profile** **a** Comparison between the total RIXS profile and the theoretical ones computed using transition dipole moments yielded by the HCH, FCH and XCH methods. Total and partial theoretical RIXS cross-sections for different local structure subsets computed with XCH **b** and HCH **c** dipoles. The percentages show the contributions of D1 and D2 structures in the total RIXS profile. Different quantum chemical methods result in different numbers because they give different transition dipole moments. We omit here the FCH partial analysis because its almost identical to the XCH case shown in panel **b**.
